# Gonococcal peritonitis diagnosed post laparotomy in a 38-year-old woman: a case report

**DOI:** 10.4076/1757-1626-2-8080

**Published:** 2009-09-17

**Authors:** Stephanie MS Wilmore, Carl J Reynolds

**Affiliations:** 1 Department of Surgery, Chase Farm Hospital, Enfield, Middlesex, EN2 8JL, England; 2 Department of Surgery, Basildon and Thurrock University Hospital, Nethermayne, Basildon, SS16 5NL, England

## Abstract

A 38-year-old South African lady with a background history of cervical intra-epithelial neoplasia and recent colposcopy presented to the emergency department with severe abdominal pain. Initial investigations were non-diagnostic and despite empirical antibiotic therapy the patient developed peritonism. Post-laprotomy the diagnosis of severe pelvic inflammatory disease and peritonitis secondary to infection with Neisseria Gonorrhoea was made.

## Case presentation

A 38-year-old white South African female presented to the Emergency department complaining of an 8 hour history of sudden onset generalized, severe abdominal pain associated with very liquid green-brown stools.

The pain was colicky in nature and there were no alleviating or exacerbating factors. Frequency of passing stool was increased from one stool a day to six stools in the previous 12 hours. There was no blood passed per rectum. There was no nausea, vomiting, or dysuria.

There was no previous history of abdominal pain and no recent history of weight loss, anaemia, jaundice, or lethargy. There were no risk factors for food poisoning and all contacts were well.

One year prior to presentation the patient recalled noticing an odourless mucous-like discharge present at the beginning and end of her period.

Four months prior to presentation the patient had visited family in South Africa. She stayed in an air conditioned house and did not visit any malaria endemic regions.

Three months previously a routine smear test had shown moderate dyskaryosis. Colposcopy and biopsy were performed and the patient was diagnosed with cervical intra-epithelial neoplasia grade III (CIN III). The patient was nulliparous, had a regular 28 day menstrual cycle, and denied abnormal bleeding and dysparunia.

There was no other previous medical or surgical history and the patient took no regular medications but was penicillin allergic, reporting previous penicillin associated breathlessness and rash.

There was no significant family history. The patient lived with her long term male partner in a house and worked as a secretary in an office. She had never smoked. She drank moderately consuming approximately six units of alcohol per week. She denied intravenous drugs use and had had unprotected sexual intercourse only with her long term partner for the past two years.

On examination, the patient was in visible discomfort. There was no scleral yellowing or conjunctival pallor. There were no stigmata of chronic liver disease. She was pyrexial (38 degrees) tachycardic (100 beats per minute), tachypnoeic (26 breaths per minute) and hypotensive (97/64 mmHg). The abdomen was distended and tender to palpation. There was no guarding or rigidity. There was no organomegaly or shifting dullness and bowel sounds were present and hyperactive. Digital rectal examination was unremarkable.

Initial investigations showed showed hematuria (3+), proteinuria (1+), ketonuria (1+) and leukocytouria done with dipstick method. Urine test for pregnancy was negative. Microscopy and culture of blood, urine and stool were negative. Test for HIV (ELISA) was also negative. Arterial blood gas analysis showed pH - 7.32, pCO2 - 4.4 kPa, pO2 - 9.1 kPa, HCO3 - 17 mmol/L, and BE -8.2 mmol/L. Hemogram revealed Hb of 14.7 g/dL, WBC 11.5 × 10^9^/L with neutrophils 9 × 10^9^/L. Urea, electrolytes, liver function tests, and amylase were normal. C-reactive protein was elevated (138.2 mg/L; normal range < 10 mg/L).

An abdominal and erect chest X- Rays were normal.

The differential diagnosis at this point was:

â€¢Â acute bacterial or viral gastroenteritis

â€¢Â appendicitis

â€¢Â perforated bowel (peptic ulcer or appendix) leading to secondary bacterial peritonitis

â€¢Â first presentation of inflammatory bowel disease

The patient was given a stat dose of Clindamycin 600 mg IV followed by the same dose IV qds, as well as Gentamicin 7 mg/kg once a day intravenously. Her condition deteriorated over the next 4 days as her abdominal pain increased in severity, she remained pyrexial, and required fluid resuscitation to support her blood pressure.

On day 4 a computed tomography (CT) scan was performed which revealed dilated loops of proximal small bowel with significant wall thickening. One of the loops showed irregularity of the lumen. There was a small amount of free fluid in the pelvis but no significant abnormality in the upper abdominal viscera. There was no evidence of acute cholecystitis. The right iliac fossa appeared normal. There was no free intraperitoneal air.

On day 5 the patient developed peritonism, she was lying still with a positive cough test, rebound and percussion tenderness, board-like abdominal rigidity, guarding and absent bowel sounds, and an emergency laprotomy was performed.

Operative findings were notable for the identification of free non-malodorous pus in the peritoneum (subphrenic, pelvic and in both paracolic gutters). The totality of the bowel from stomach to rectum was carefully scrutinised for a perforation but was normal, including the appendix. The liver, gallbladder, ovaries and uterus appeared normal. No identifiable cause was found for pus origin.

After discussion between clinicians and microbiologist and in view of her past gynaecological history, nucleic acid amplification tests (NAATs) were performed for Neisseria gonorrhoea and Chlamydia trachomatis on operative culture media [1]. NAATs were positive for the former but negative for the latter. The diagnosis of severe pelvic inflammatory disease was made. In view of this, together with the poor response to Clindamycin and Gentamycin, antibiotic therapy was changed to Ceftriaxone 2 g IV bd, Metronidazole 500 mg IV tds, and 250 mg stat PO of Doxycycline followed by 100 mg PO od.

She was admitted to intensive therapy unit (ITU) post operatively. In the high dependency unit (HDU) her recovery was complicated by further intense abdominal pain due to a paralytic ileus which resolved by day 8.

## Discussion

Our case is strikingly similar to a number of other cases reported recently in the literature [2]-[4]. In each case a woman aged over 30 presents as a surgical emergency with an acute abdomen and diagnosis is made post-laprotomy when Neisseria gonorrhoea is isolated from purulent exudate in the peritoneal cavity. The possible significance of age related endocervical changes predisposing to this presentation is discussed by Brunel et al [2].

Neisseria gonorrhoea is the second most common sexually transmitted infection in the United Kingdom and there are reports that its prevalence is increasing [4,5]. Infection at the endocervix can give rise to increased or altered vaginal discharge, this is the most common symptom (up to 50% of cases), but it is also frequently asymptomatic in females (up to 50% of cases) as opposed to males [5,6]. Untreated, gonorrhoea can lead to serious complications such as pelvic inflammatory disease (PID) in women, in turn causing possible scarring of the upper reproductive tract, infertility and ectopic pregnancies. Disseminated gonococcal infection, resulting from bacteraemia, may result in arthritis, endocarditis, skin infections and meningitis.

## Abbreviations

CIN III: cervical intra-epithelial neoplasia grade III; NAATS: nucleic acid amplification tests; PID: pelvic inflammatory disease.

## Consent

Written informed consent was obtained from the patient for publication of this case report and accompanying images. A copy of the written consent is available for review by the Editor-in-Chief of this journal.

## Competing interests

The authors declare that they have no competing interests.

## Authors' contributions

SMS Wilmore analysed and interpreted the clinical aspects of the case. CJ Reynolds carried out a literature review, wrote the discussion, and revised the whole manuscript.

All authors read and approved the final manuscript.
